# A promising randomized trial of a new therapy for obsessive–compulsive disorder

**DOI:** 10.1002/brb3.67

**Published:** 2012-06-26

**Authors:** Xian-Zhang Hu, You-Sheng Wen, Jian-Dong Ma, Dong-Ming Han, Yu-Xia Li, Shu-Fan Wang

**Affiliations:** 1 The Second Affiliated Hospital of Xinxiang Medical UniversityXinxiang City, 453002, Henan Province, China; 2 Wuhan Mental Health Center (Wuhan Hospital for Psychotherapy), Tongji Medical College of Huazhong University of Science and TechniqueWuhan City, Hubei Province, China; 3 The First Affiliated Hospital of Xinxiang medical UniversityXinxiang City, 453002, Henan Province, China; 4 Zhengzhou Hospital of Traditional Chinese MedicineZhengzhou City, 450007, Henan Province, China

**Keywords:** Cognitive–behavioral therapy, cognitive–coping therapy, OCD, remission, response

## Abstract

Pharmacotherapy and cognitive–behavioral therapy (CBT) are currently the most effective interventions for treating obsessive–compulsive disorder (OCD). These treatments, however, are time consuming and in some cases the patients do not show significant improvement. In all, 30%–60% of OCD patients do not respond adequately to pharmacotherapy and 20%–40% of OCD patients who complete CBT do not improve significantly, suggesting a more efficacious approach is needed. The objectives of this study are to demonstrate an efficacious pharmacotherapy plus psychotherapy, named cognitive–coping therapy (CCT), for OCD and to investigate the efficacy of this approach in a larger sample size. Therefore, a total of 108 patients with OCD were randomly allocated into three groups: pharmacotherapy (*N* = 38), pharmacotherapy plus CBT (PCBT, *N* = 34), and pharmacotherapy plus CCT (PCCT, *N* = 36). The severity of symptoms and the patients' functioning were assessed pretreatment and after 7, 14, 21 days, and 1-, 3-, 6-, and 12-month treatment using the Yale-Brown Obsessive Compulsive Scale and Global Assessment of Functioning (GAF). Compared with the pharmacotherapy and PCBT groups, the severity of OCD symptoms was significantly reduced (*P* < 0.001), the rates of response (100%) and remission (85.0%) were significantly higher (*P* < 0.001), and relapse rate was lower (*P* = 0.017) in PCCT group during the 1-year follow-up. In addition, the GAF score was significantly higher in the PCCT group than in the other two groups (*P* < 0.001). Our preliminary data suggest that PCCT is a more efficacious psychotherapy for OCD patients than pharmacotherapy or PCBT.

## Introduction

Pharmacotherapy and cognitive–behavioral therapy (CBT) are major treatment options for obsessive–compulsive disorder (OCD). Although these treatments have been continuously improved for several decades, there are still limitations (Taylor 2005; Bonchek 2009; Maher et al. 2010). It takes more than 12 weeks of pharmacotherapy to obtain significant clinical response (Greist et al. 1995). Up to 60% of OCD patients do not respond adequately to pharmacotherapy and are considered to be resistant to pharmacotherapy (Bjorgvinsson et al. 2007). Controlled trials combining pharmacotherapy with CBT demonstrate no clear advantage over CBT alone (Cottraux et al. 1990; Foa et al. 2005; Sousa et al. 2006). CBT for OCD, an exposure-based strategy integrated with cognitive therapy, usually takes 14–20 weeks and emphasizes education about anxiety psychopathology and repeated exposure to fear-eliciting cues (March and Mulle 1998). It is often used in combination with restructuring of false threat appraisals (Westra and Stewart 1998; Clark 2005). Although CBT can reduce 48% of symptoms in adult OCD patients (Abramowitz et al. 2002), up to 40% of OCD patients who complete CBT do not significantly improve or respond to treatment (Stanley and Turner 1995; Whittal et al. 2005). In all, 50%–75% of patients remain symptomatic following a full treatment course (de Haan 2006). Twenty percent to 30% of patients refuse to enter or drop out of CBT (Foa et al. 1983, 2005; Abramowitz 1997). OCD symptoms usually require up to 12–20 weeks of treatment with standard, weekly CBT to show a clinical response. Cognitive therapy in CBT for OCD is no more effective than exposure and response prevention (ERP; Abramowitz et al. 2005). Subsequently, many efforts have been made to complement CBT (Schwartz and Beyette 1997; Twohig et al. 2006; Coelho et al. 2007; Fairfax 2008; Hanstede et al. 2008; Bonchek 2009; Brown and Hooper 2009).

CBT is still in the process of improvement (Taylor 2005; Turner 2006). CBT asserts that OCD is caused when intrusive thoughts (obsessive thoughts, images, urges, or doubts) are falsely appraised as an indicator of significantly negative events for the individual or the individual's loved ones. The OCD patient seeks to prevent the imagined dreaded outcomes or escalating states of anxiety through their compulsions (Rachman 1998; Salkovskis 1999; Clark 2004, 2005). This theory, however, does not fully explain why some people appraise intrusive thoughts as an indicator of negative events while others do not. CBT asserts that once intrusive thoughts are perceived as non-threatening, the obsessive thoughts and the compulsions can be eliminated (Abramowitz et al. 2005; Clark 2005). In clinical practice, CBT encourages OCD patients to refrain from compulsions via ERP whenever the obsession enters conscious awareness. Furthermore, the goal of CBT is to normalize intrusive thoughts so they are no longer perceived as a highly threatening cognition (Clark 2005). Due to its reliance on ERP to reach the goal, CBT cannot benefit all those who complete treatment because some patients are unable or unwilling to tolerate the distress associated with ERP (Taylor 2005).

**Figure 1:**
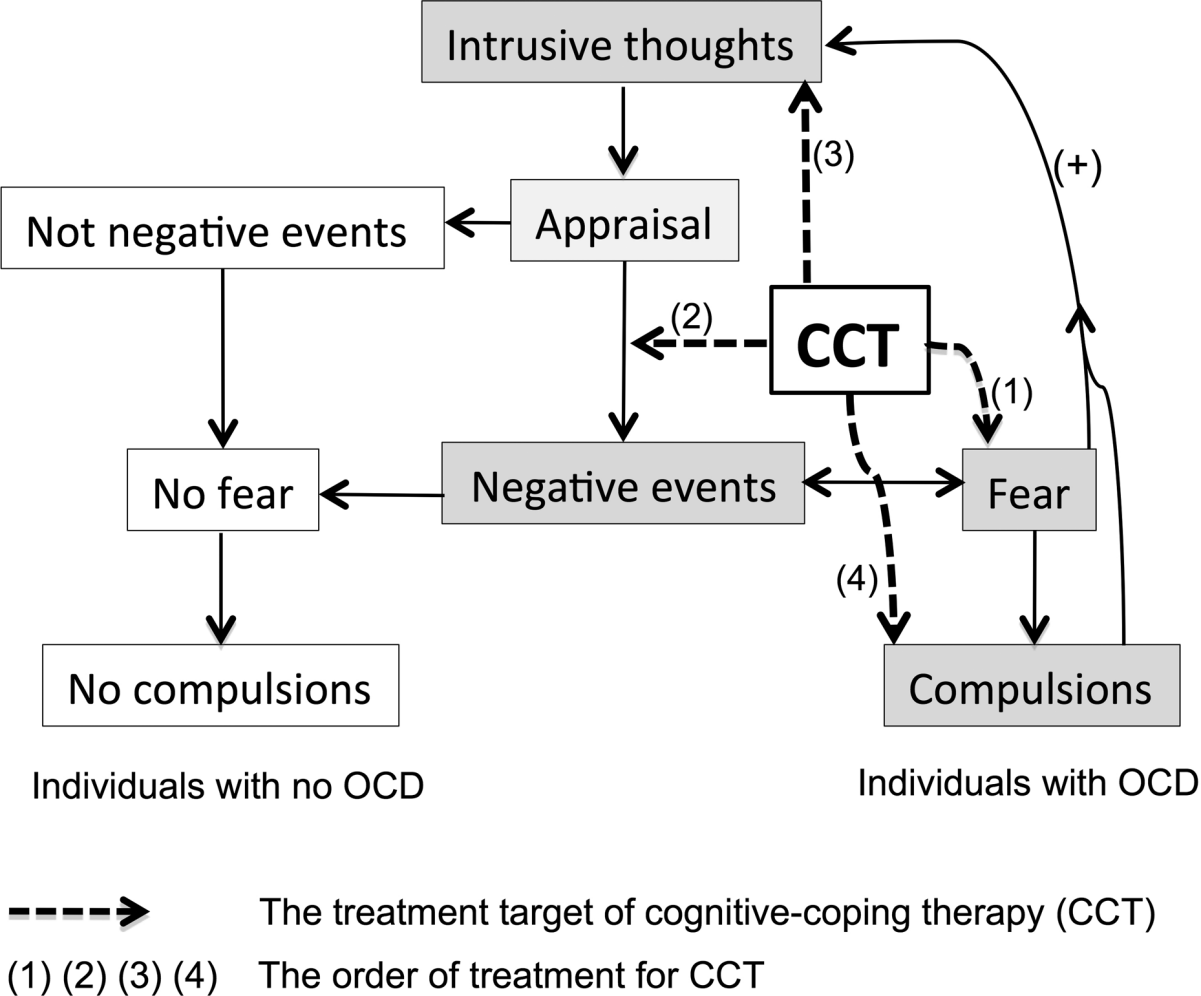
The targets of cognitive–coping therapy (CCT). Intrusive thoughts are considered stimuli. After appraising the stimuli, if individuals construct a threatening/harmful meaning, both the intrusive thoughts and the threatening/harmful meaning will become stressors and induce negative mood, such as fear and anxiety, and response to stimuli will become the compulsions. Proper coping strategies can modify the way individuals think, change the threatening/harmful meaning, and relieve the negative mood and compulsions.

According to stress and coping theory, two processes, cognitive appraisal and coping, are identified as critical mediators of stressful person–environment relationships and their immediate and long-term outcomes (Folkman et al. 1986). Coping is defined as “constantly changing cognitive and behavioral efforts to manage specific external and/or internal demands that are appraised as taxing” or “exceeding the resources of the person” (Folkman and Lazarus 1980). Coping uses conscious cognitive and behavioral efforts to solve problems and minimize stress or conflict. Appraising evaluates the personal significance of one's relationships with others or the environmental and the available options for coping (Lazarus 2006). Common type of coping styles are problem-focused coping (information seeking and problem solving), emotion-focused coping (expressing emotion and regulating emotions; Lazarus and Folkman 1984), and appraisal-focused coping (denial, acceptance, social comparison, redefinition, and logical analysis; Callan and Hennessey 1989). Appraisal-focused strategies occur when individuals modify the way they think by altering goals and values. If the intrusive thoughts and the imagined outcome of negative events are considered stressors, we propose that proper coping strategies may change appraisal cognition about the stressors, the mood associated with false appraisal, and individual's responses to the stressors (compulsions) (Fig. 1).

Cognitive-coping therapy (CCT) has been developed for treating OCD and is characterized by three aspects (Hu 2010; Hu and Ma 2011). First, CCT posits that the fear of negative events should be the target of treatment. Second, CCT seeks to break the association with intrusive thoughts and the fear of negative events through appraisal-focused coping rather than normalizing the intrusive thought, as done in CBT. Third, due to CBT's reliance on ERP, CCT encourages OCD patients to use coping strategies to deal with intrusive thoughts, the fear of negative events, and compulsions (Fig. 1). OCD may be expressed as the formula: OCD ≍ intrusive thoughts_(n1)_ × false appraisal_(n2)_ × fear_(n3)_ × compulsions_(n4)_ (*n* is ≥0 integer and stands for intensity). If *n* > 0, individuals will suffer from OCD. The greater the value of *n* is, the more serious the OCD symptoms are. Should any *n* = 0, individuals will not manifest OCD symptoms. The targets of CCT are n3, n2, n1, and n4, whereas CBT mainly targets n4 by ERP and n2 by cognitive therapy (Salkovskis 1999; Fisher and Wells 2005).

Previous studies demonstrated that pharmacotherapy plus CCT (PCCT) is an efficacious approach for OCD patients (Hu and Ma 2011). In this study, we evaluate the proposal that PCCT provides OCD patients more benefits by quickly relieving OCD symptoms and significantly improving their social-occupational function in a larger sample size.

## Methods

### Participants

**Figure 2:**
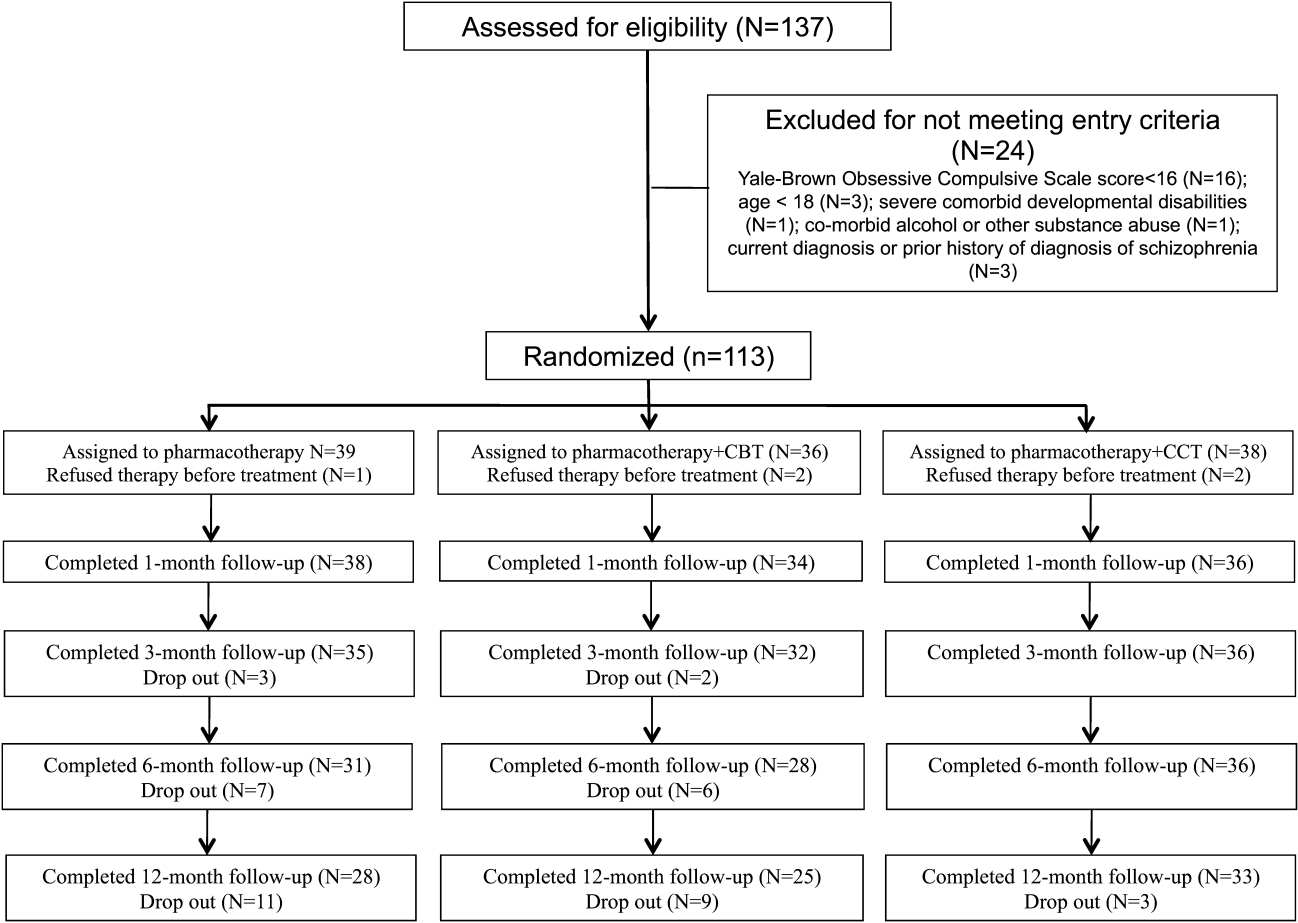
CONCORT diagram.

A total of 137 OCD patients were recruited by clinical referral in the Outpatient Department of the Second Affiliated Hospital of Xinxiang Medical University and Wuhan Mental Health Center in P. R. China from August 2008 to August 2010. All patients were Chinese Han and met the *DSM-IV-RT* diagnostic criteria for OCD. The diagnoses were made by two senior psychiatrists after face-to-face interviews according to the SCID-I/P (First et al. 2002). Total score in the Yale–Brown Obsessive Compulsive Scale Severity Rating (Y-BOCS-SR) was ≥16. Patients were excluded from the study if their age was <18 years old or if there were comorbid severe cognitive deficits or other axis I diagnosis, such as schizophrenia. Patients were not excluded for comorbid anxiety or depressed mood. All patients provided written informed consent in accordance with research guidelines for the protection of human participants from Xinxiang Medical University. Twenty-four patients were excluded and 113 were randomly assigned into three groups: pharmacotherapy (*N* = 39), pharmacotherapy plus CBT (PCBT) (*N* = 36), and PCCT (*N* = 38). Five patients declined participation because they did not want to receive any treatment (Fig. 2). One hundred and eight OCD patients were entered into the study. There was no significant difference between groups in gender distribution, marriage status, comorbidity of anxiety or depressed mood, age, age at onset, duration of illness, and the Y-BOCS-SR score among the three groups. There were no significant differences in medicine dosages among the three groups. The demographic and clinical data for the study population are shown in Table 1.

**Table 1:** Demographic and clinical characteristics of patients

	Pharmacotherapy (*N* = 38)		Pharmacotherapy +CBT (*N* = 34)		Pharmacotherapy +CCT (*N* = 36)	
			
	*N*	%	*N*	%	*N*	%
Gender						
Male	24	63.2	19	55.9	23	63.9
Female	14	36.8	15	44.1	13	36.1
Married						
Yes	22	58.0	21	61.8	12	58.3
No	15	42.0	13	38.2	15	41.7
Obsessions						
Contamination (e.g., dirt and germs)	22	57.9	21	61.6	22	61.1
Aggressive (e.g., harming others)	5	13.2	8	23.5	7	19.4
Sexual (e.g., image of incest)	0	0.0	1	2.9	1	2.8
Religious (e.g., blasphemous thoughts)	2	5.3	2	5.9	3	8.3
Hoarding (e.g., fears of discarding paper)	2	5.3	1	2.9	2	5.5
Pathological doubt	8	21.1	11	32.3	9	25.0
Symmetry or exactness (e.g., books aligned imperfectly)	10	26.3	9	26.4	9	25.0
Other (luck number, image, thought)	10	26.3	7	20.6	8	22.2
Compulsions						
Washing (e.g., hands, excessive showering)	22	57.9	21	61.6	22	61.1
Checking (e.g., locks, schoolwork)	10	26.3	12	35.3	12	33.3
Repeating (e.g., routine actions, steps)	11	28.9	10	29.4	12	33.3
Hoarding (e.g., papers, trash)	8	21.1	8	23.5	8	22.2
Ordering/Arranging-1 (e.g., books, clothes)	7	25.0	6	17.6	8	22.2
Ordering/Arranging-1 (e.g., mental ritual, counting, touch)	5	13.2	7	20.6	5	13.9

	Mean	SD	Mean	SD	Mean	SD
Age	27.7	9.3	28.9	11.2	28.1	8.2
Age at onset	19.8	5.2	23.0	9.9	21.3	7.0
Duration of illness (years)	5.8	2.8	5.5	5.4	6.6	4.6
Pretreatment Y-BOCS-SR	25.6	5.5	25.1	5.8	26.4	6.5
Pharmacotherapy						
Chlorimipramine	143.0	46.5	139.9	48.8	141.6	47.3
Paroxetine	23.6	8.3	23.1	7.8	23.4	7.4

### Treatments

To achieve maximum benefit, we did not designate placebo and CCT only. Medication for all patients was chlorimipramine (100–250 mg/day). After six weeks patients were administered chlorimipramine in combination with paroxetine (20–40 mg/day; Yuan et al. 2006) if they could not tolerate the side effects of the higher dosage of chlorimipramine or if they did not benefit from only chlorimipramine (>150 mg/day). Medications were prescribed for the patients by the psychiatrists, who were not involved in the psychological therapy. The CBT therapist and the CCT therapist were blinded to each other and did not participate in the pharmacotherapy.

Patients undergoing CBT received 14 weekly 60- to 120-min sessions in accordance with the CBT guide (Clark 2004), and then one or two phone calls monthly for nine months. CBT consisted of cognitive techniques as well as ERP with homework exercises. Although formal cognitive therapy procedures were not used, dysfunctional cognitions were discussed within the context of exposure. ERP involved graded exposures to both imagined and real situations that provoked compulsions, accompanied by prevention of compulsions or avoidance. Both in vivo and imagining exposures were conducted, during which patients faced their fears for a prolonged period of time without ritualizing. Patients were asked to stop ritualizing after the first exposure session. In addition to their ERP sessions with the therapist, patients were assigned at least 1 h of ERP homework daily and were asked to record any rituals. The CBT therapists were trained and licensed in the Chinese–German CBT training center in Wuhan City, Hubei Province, P. R. China.

In this study, patients had been diagnosed before undergoing the treatments.

CCT has been described in Chinese (Hu 2010; Hu and Ma 2011). Briefly, CCT consists of four therapeutic steps in each session and one step of relapse prevention (also see Fig. 1).

Step 1. *Information collecting*. Collecting enough clinical information and establishing a good therapeutic alliance were two major goals in this step. The purpose of information collection was to clarify the main problems, including the clinical manifestations and/or any obstacles that the patients encountered during the treatment.

Step 2. *Identifying and coping with fear*. The first goal in this step was to use discussion to help patients identify their fear and its role in the onset of OCD. In CCT, fear was considered an important factor in the onset of OCD. In essence, OCD patients usually feared that negative events (e.g., death) will happen to them or their loved ones. It was the fear of imagined, dreaded negative events that invoked anxiety and resulted in the neutralizing or avoidance behaviors (compulsions). Discussing in detail the role of fear helps patients prepare to cope with fear. The second goal is to reduce the extent of fear, using appraisal-focused coping strategies (e.g., rational or denial) to cope with the imagined negative events.

Step 3. *Coping with intrusive thoughts*. First, it was necessary to identify the roles of intrusive thoughts in the causation of OCD. In CCT, intrusive thoughts symbolized the imaged, dreaded negative event the OCD patient feared. Second, using cognitive reconstruction, the therapist helped the patient recognize there was no association between intrusive thoughts and the negative events. Third, the therapist encouraged the patient to cope with intrusive thoughts by using appraisal-focused coping strategies, such as acceptance, ignorance, and/or sublimation. The goal was to teach patients to allow intrusive thoughts to exist in their minds, pay no attention to them, ignore them, and experience meaningful daily activities by practicing proper coping strategies, instead of ERP of CBT.

Step 4. *Coping with compulsions*. The goal in this step was to eliminate compulsions. When steps 2 and 3 were completed successfully, patients would understand it was unnecessary to neutralize the fear. Thus, it would be easier for patients to cope with and to avoid the compulsions. Patients with OCD were encouraged to practice using proper coping strategies in the therapy room. During this process, it was very important to avoid using ERP.

In each therapy session, patients moved through all four steps. When the OCD symptoms were eliminated after several sessions, the patient habitually urged to check if the intrusive thoughts were still in their mind, which generally resulted in the immediate emergence of intrusive thoughts. To prevent relapse, the therapist reminded the patient that the urge to check should be considered an intrusive thought to be coped with.

Patients undergoing PCCT received 14 weekly 40- to 60-min sessions of CCT and then one or two phone calls monthly for nine months.

Patients undergoing pharmacotherapy received one or two phone calls monthly for nine months after the 14-week treatment period.

### Assessments

Y-BOCS-SR and Global Assessment of Functioning (GAF) were assessed before treatment and after 7, 14, 21 days, and 1, 3, 6, and 12 months of treatment. Y-BOCS was translated into Chinese with high reliability and validity in 1996 (Zhang et al. 1996) and widely used. GAF proved to be a reliable and valid measurement of social–occupational–psychological functioning (Jones et al. 1995; Soderberg et al. 2005).

Y-BOCS-SR was also used to assess prognosis. Many studies accepted a lower standard of ≥25% decrease in Y-BOCS-SR as a response to treatment (Hollander et al. 2010). In this study, response to treatment was defined as a 35% decrease in Y-BOCS-SR score from baseline. Remission was defined as ≥80% decrease in Y-BOCS-SR score from baseline. Relapse was defined as loss of responder status for longer than 2 weeks (Foa and Kozak 1996).

The OCD residual symptoms were self-rated by patients. A set of consecutive integers from 0 to 100% was given to patients who were directed to indicate by what percentage symptoms were improving. A score of 0% meant symptoms were completely relieved; a score of 100% meant the severity of symptoms had not changed after treatment compared with pretreatment. Patients could produce scores greater than 100% if the symptoms became more serious after treatment.

The GAF scale was used to access an individual's overall level of functioning in carrying out daily activities as a predictor of treatment outcome. GAF is a continuous measure (from 0 to 100) used by clinicians and physicians to subjectively rate social, occupational, and psychological functioning (Schorre and Vandvik 2004). The GAF is constructed as an overall measure of how patients are doing and rates psychological, social, and occupational functioning, covering the range from positive mental health to severe psychopathology (Aas 2011). Despite its widespread use, the GAF may have its limitations. One major limitation of GAF is that it combines several dimensions of psychopathology on a single 100-point scale, specifically combining symptoms and social, occupational functioning (Goldman 2005). To solve the problem, we rated the GAF separately and focused primarily on the social and occupational functioning. Although GAF can be valued either as a single score (the severe of the symptom and functioning) or separate scores for symptoms and functioning (Aas 2011), when the GAF is scored separately, it can be scored reliably (Niv et al. 2007).

### Quality control

Therapists providing CBT received training and supervision before participating in the study. Training included procedure review and completion of at least one training case under supervision. During the study, weekly group supervision for each treatment was held. Only experienced therapists, who displayed excellent protocol adherence in the training program, were involved in the study. Therapist providing CCT followed the procedure described above. Both lively supervision and taped supervision for CCT were held weekly.

Two trained psychiatrists who had no other contact with participants evaluated all assessments. The inter-rater reliability was high enough for the study (*r* > 0.95, *P* < 0.001). OCD symptoms were recorded using the Y-BOCS Symptom Checklist (Y-BOCS-SC; Goodman et al. 1989).

### Statistical analysis

Analysis of covariance (ANCOVA) with repeated measured and baseline data control and Tukey Honest Significant Difference (HSD) post hoc were performed to test the effects of treatment, time, and interaction on OCD Y-BOCS-SR score and GAF score, using the SAS (ver 9.1)'. The Tukey HSD is the most widely used post hoc test in psychological and the behavioral sciences (http://www.une.edu.au/WebStat/unit_materials/c7_anova/oneway_post_hoc.htm). Chi-square was performed to analyze the response rate and clinical remission rate. When this requirement was not met for a 2 × 2 table, a Fisher Exact Probability Test was performed. Linear regression analyses were performed to measure the correlation between the reduction in Y-BOCS-SR score rated by psychiatrists and the improvement of the OCD symptoms rated by patient self-report to confirm the accuracy of the rating. Multiple linear regression was performed to investigate correlative factors that influenced the efficacy of treatment.

An intent-to-treat (ITT) analysis using the last observation carried forward (LOCF) was conducted to examine all participants who received treatment for any time period.

## Results

### Evaluation reliability on the severity of OCD symptoms

**Figure 3:**
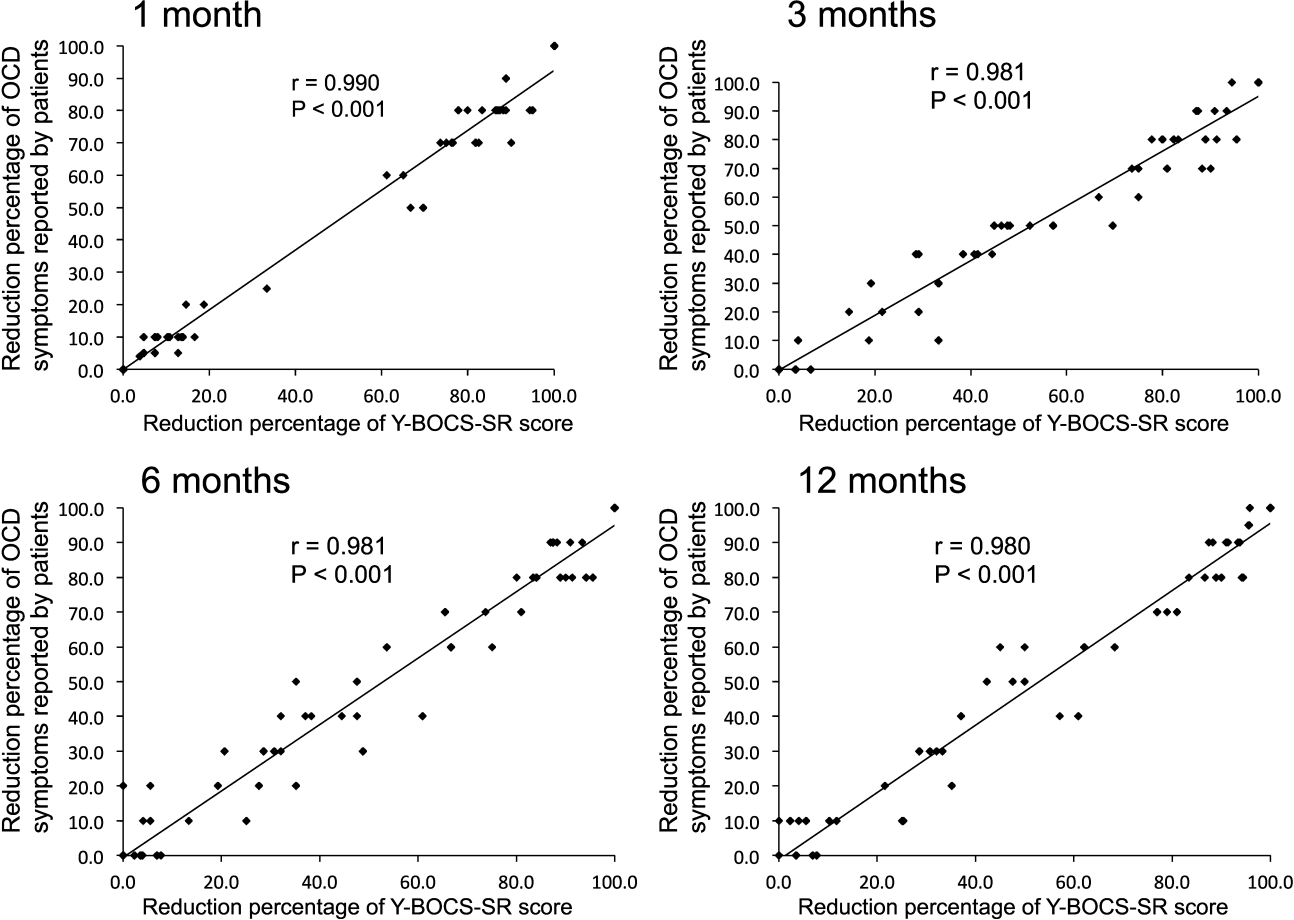
Reliability analysis on the severity of OCD symptoms.

Reliability was detected using linear regressive analysis between the variables of reduction percentage of Y-BOCS-SR score and the reduction percentage of OCD symptoms at the four time-points after treatment. The correlation coefficients were greater than 0.98 (*P* < 0.001) (Fig. 3).

### Changes in the severity of OCD symptoms

ANCOVA analysis showed that the reduction in Y-BOCS-SR total scores was significantly greater overall, dependant on the treatment and treatment period (*F* = 3.66, df1 = 14, df2 = 328, *P* < 0.001). The repeated measures analysis of variance showed that the interaction of treatment and treatment period resulted in a reduction in Y-BOCS-SR total scores (*P* < 0.001).

The ANOVA post hoc tests showed that the Y-BOCS-SR scores were not different among the three groups (*P* > 0.05). Compared with baseline, the Y-BOCS-SR score was significantly reduced only in the PCCT group at month 1 (*P* < 0.001). The score was significantly reduced in the PCCT group (*P* < 0.001) and the PCBT group (*P* < 0.05) at months 3, 6, and 12. In the pharmacotherapy group, there was a trend of significant reduction at month 6 (*P* = 0.059) and a significant reduction at month 12 (*P* < 0.05), but no differences were found at months 1 and 3 (*P* > 0.05) (Table 2).

**Table 2:** The OCD symptom severity changes after treatment

	Y-BOCS-SR^1^		Response rate^2^		Remission rate^3^		GAF score^d^	
				
	Mean	SD	*N*	%	*N*	%	Mean	SD
Pharmacotherapy								
Pre-treat (*n* = 38)	25.6	5.5					52.3	5.3
1 month (*n* = 38)	25.4	4.9	0	00	0	0.0	52.9	5.5
3 months (*n* = 35)	23.6	5.3	14	40.0	0	0.0	60.0	10.5
6 months (*n* = 31)	23.0	5.7	16	51.6	2	6.5	60.5	10.1
12 months (*n* = 28)	22.4	5.3	13	46.4	1	3.1	62.2	8.5
Pharmacotherapy + CBT								
Pre-treat (*n* = 34)	25.1	5.8					49.7	4.5
1 month (*n* = 34)	24.7	5.5	0	0.0	1	2.9	51.8	5.1
3 months (*n* = 32)	21.1	6.2	17	53.1	2	6.3	59.6	8.5
6 months (*n* = 28)	19.3	6.8	16	57.1	5	17.8	64.1	10.4
12 months (*n* = 25)	17.6	7.0	17	68.0	5	20.0	67.6	12.8
Pharmacotherapy + CCT								
Pre-treat (*n* = 36)	26.4	6.5					49.4	7.3
1 month (*n* = 36)	6.2	3.3	36	100.0	23	63.9	85.9	9.9
3 months (*n* = 36)	4.5	3.2	36	100.0	30	83.3	90.5	12.9
6 months (*n* = 36)	4.0	2.1	36	100.0	31	86.1	93.4	10.4
12 months (*n* = 33)	4.5	5.6	33	100.0	30	83.1	92.0	13.7

a The Y-BOCS-SR score. The Y-BOCS-SR score in PCCT were significantly lower than those in pharmacotherapy only and PCBT at any time-point after treatment (*P* < 0.001).

b The response rates in PCCT were significantly higher than those in pharmacotherapy only and PCBT at all time-points after treatment (*P* < 0.001), respectively. There were no differences in response rates between pharmacotherapy and PCBT at any time-points.

c The remission rates in PCCT were significantly higher than those in pharmacotherapy only and PCBT at all time-points after treatment (*P* < 0.0001). There were no differences in remission rates between pharmacotherapy and PCBT at any time-points.

Among the three groups, the Y-BOCS-SR scores were significantly lower in the PCCT group than in the pharmacotherapy and PCBT groups at any time-point after treatment (*P* < 0.001). The score at month 6 and 12 in the PCBT group was significantly lower than in the pharmacotherapy group (*P* < 0.05 and *P* < 0.01), respectively.

The response rate in the PCCT group was 100% at months 1, 3, 6, and 12, significantly greater than the pharmacotherapy group or PCBT group (Fisher's exact test, *P* < 0.001) (Table 2). No significant differences in response rates between the pharmacotherapy group and PCBT group were found at any time-point, although there was a trend difference between the two groups at month 12 (Table 2).

Remission rates were higher in the PCCT group (≥63.9%) than in pharmacotherapy group or PCBT group at month 1, 3, 6, and 12, respectively (*P* < 0.0001; Table 2). There was no significantly different remission rate between the PCBT group and the pharmacotherapy group (Fisher's exact test, *P* > 0.05) (Table 2).

### The social-occupational functioning

ANCOVA analysis showed that GAF was significantly different overall (*P* < 0.001). The GAF score showed a significantly greater increase in the PCCT (*P* < 0.001) group than in the pharmacotherapy group and PCBT group over the treatment time. The repeated measures analysis of variance showed that the interaction of treatments and time significantly affected the GAF score (*P* < 0.0001).

The ANOVA post hoc tests showed that there was no difference in the GAF scores before treatment among the three groups (*P* > 0.05). Compared with the baseline, the average GAF score was significantly increased at month 1 in the PCCT group (*P* < 0.001) and remained at a significantly higher level at months 3, 6, and 12. At months 1, 3, 6, and 12, the GAF score was higher in the PCCT group than in the pharmacotherapy group and PCBT group (*P* < 0.001) (Table 2). At month 3, the GAF score increased in the pharmacotherapy group and in the PCBT group (*P* < 0.001) when compared with the baseline.

### Factors correlated with the efficacy of PCCT

Multiple linear regression analysis was performed using the Y-BOCS-SR score as a dependent variable, and gender, education (year), duration of OCD, severity of symptom, and insight as independent variables to investigate the correlated factors with the efficacy of PCCT. The results show that only insight entered the formula at week 2 (*R*^2^ = 0.52, *P* = 0.025), week 4 (*R*^2^ = 0.59, *P* = 0.025), month 3 (*R*^2^ = 0.76, *P* = 0.001), month 6 (*R*^2^ = 0.70, *P* = 0.003), and month 12 (*R*^2^ = 0.64, *P* = 0.007), respectively.

### Relapse rates and ITT

During follow-up, there were 15 (39.5%) participants in the pharmacotherapy group, 18 (52.9%) in the PCBT, and 36 (100%) in the PCCT that initially responded to the treatments, but 8 (53.3%) in the pharmacotherapy group, 6 (33.3%) in the PCBT, and 2 (5.6%) in the PCCT group relapsed (Fisher exact test, *P* < 0.001). There was no significant difference between pharmacotherapy and PCBT (χ^2^ = 1.34, df = 1, *P* = 0.124).

After the 12 months follow-up, 11 (28.2%) participants dropped out in the pharmacotherapy group and 9 (25.0%) dropped out in the PCBT group. Three (7.9%) participants dropped out of the PCCT (Fisher's exact test, *P* < 0.05). Using LOCF to examine all participants for ITT analysis; results were similar to those described above for the severity changes of OCD symptoms and social–occupational function in the three groups.

## Discussion

Our findings demonstrate that PCCT can be used to treat most OCD symptoms with better compliance, higher response and remission rates, and reduced OCD symptom severity quickly, and with improved social–occupational function in OCD patients. The insight of OCD patients may be a predictor of the outcome of PCCT.

Our study also indicates that PCCT takes significantly less time to relieve OCD symptoms. Previous data showed that up to 12 weeks of treatment were required to see a response in OCD symptoms to medication or CBT, and even CBT combined with pharmacotherapy (Salkovskis 1999; Simpson et al. 2008). Several factors might be responsible for why PCCT treats OCD quickly, although none are supported with direct evidence. First, CCT may play a pivotal role in PCCT because response to pharmacotherapy is usually delayed and takes up to 8–12 weeks (Greist et al. 1995; Math and Janardhan Reddy 2007). Our data indicate that the response to PCCT is significantly shorter (<1 month) than pharmacotherapy only. Second, CCT may set a proper therapeutic target order (from fear to intrusive thoughts and then to compulsions). Third, coping skills may be proper strategies in CCT for OCD treatment. Fourth, according to the model of Goldapple and colleagues (Goldapple et al. 2004), four important components can be identified: intrusive thoughts, false appraisal, fear of negative events, and compulsions (Fig. 1). Individuals with false appraisal tend to believe intrusive thoughts are related to negative events and feel fear. Fear of negative events motivates an individual's neutralizing behavior (compulsions). Therefore, false appraisal and fear of negative events play important roles in the onset of OCD and can be the main targets of CCT. The intrusive thoughts themselves are indicators of negative events for individuals. In CCT, compulsions will be eliminated after the intrusive thoughts are properly coped with as stressors and are isolated from negative events. Also, the compulsions can make intrusive thoughts become more frequent, repetitive and disturbing (Clark 2005). One goal of CCT is to break down the reinforcing relationships between intrusive thoughts, negative events, and compulsions, which is achieved by using appraisal-focused and problem-focused coping strategies, instead of ERP of CBT.

PCBT was less efficacious than PCCT, but its response rate (53%–68%) is higher than pharmacotherapy alone (40%–51%). The response rate of PCBT reported in this study was lower than previous reports on CBT efficacy (Cottraux et al. 1990; Stanley and Turner 1995; Foa et al. 2005; Sousa et al. 2006; Simpson et al. 2008; Maher et al. 2010). Also, after 10–12 weeks of treatment, pharmacotherapy only treatment has a lower response rate than the 40%–60% seen in OCD pharmacologic trials (Greist et al. 1995). The lower response rates may be related to the different standard. Simpson et al. (2008) report that 74% of patients receiving pharmacotherapy plus CBT achieved responder status when defining the response as a ≥25% reduction in Y-BOCS-SR, which is lower than ≥35% reduction in Y-BOCS-SR in this study. The other possible reason may be associated with the low dosage of medicine. The dosage of chlorimipramine (average: around 140.0 mg/day) is lower than the recommended (150–250 mg/day; The Clomipramine Collaborative Study Group 1991; Math and Janardhan Reddy 2007). The dose of SSRIs (average: around 23.4 mg/day) taken by patients is also relatively lower (Stein et al. 2007). The lower dosage of medication might be associated with the lower response rate in the pharmacotherapy group (Pallanti et al. 2002; Landeros-Weisenberger et al. 2009). CBT has been devised and consistently developed for OCD treatment in Western culture since the 1960s and 1970s (Taylor 2005; Foa 2010) and was introduced to China. The response rate in this study is similar to reported studies in Western populations, suggesting that CBT is applicable in different cultures, although the efficacy of psychotherapy is affected by cultural factors (Bhui and Morgan 2007). It is known that symptoms of OCD have varied little over time (pathological scrupulosity, for example, has long been documented) or place (similar symptoms are seen across many cultures; Ames et al. 1994; Lawrence 2000). Therefore, CCT may be an applicable therapy and is worth exploring in different cultures.

CCT is closely related to, but not the same as, CBT. First, the treatment order of CCT is fear, obsessions, and then compulsions. CBT is based on the assertion that refrain the compulsions when exposure can normalize the intrusive thoughts so that it is no longer viewed as a highly threatening cognition (Clark 2005). Second, regarding goals of treatment, CCT help OCD patients cope with intrusive thoughts because more than 90% of the general population have ever experienced intrusive thoughts (O'Neill et al. 2009), while CBT targets to normalize intrusive thoughts (Clark 2005). Third, CCT emphasizes that the fear of negative events plays an important role in the onset of OCD. A crucial step of CCT is to reduce fear with coping strategies. Fourth, our preliminary data suggest that the cognitive therapy in CCT is efficacious, whereas according to Abramowitz and his colleagues (2005), the cognitive therapy in CBT is no more effective than ERP. Fifth, CCT for OCD teaches patients to use coping strategies, whereas CBT mainly uses ERP as a therapeutic strategy (Salkovskis 1985).

Also, the term “acceptance” used in CCT is not necessarily the same concept as acceptance used in acceptance and commitment therapy (ACT). First, acceptance in CCT is based on the re-explanation of the onset of OCD, which emphasizes the role of fear. Acceptance in ACT is rooted in the pragmatic philosophy of functional contextualism and is a mindfulness-based behavioral therapy that challenges the ground rules of most Western psychology (Harris 2006; Hayes et al. 2006). Second, in CCT, acceptance is defined as a coping strategy. Obsessions and fear are allowed to exist in the mind. In ACT, acceptance is taught to patients as an alternative to experiential avoidance and is not an end in itself. Rather acceptance is fostered as a method of increasing values-based action (Harris 2006). Third, the goal of acceptance in CCT is to cope with obsessions and fear. The goal of acceptance with ACT is to create a rich and meaningful life while accepting obsessions that inevitably go with life (Harris 2006; Hayes et al. 2006).

A treatment with four steps, by Dr. Schwartz, named “cognitive–biobehavioral self-treatment” or the Four-Step Self-Treatment Method, describes how knowledge about the biological basis of OCD helps patients control their anxious responses and increases their ability to resist the symptoms of OCD. Cognitive–biobehavioral treatment differs from classic ERP in one important way: the four steps enhance clients' ability to do ERP without a therapist's presence (Schwartz and Beyette 1997). Therefore, the four steps can be considered a modified CBT with core therapeutic strategy of ERP, whereas CCT uses coping strategies rather than ERP. The four steps emphasizes that OCD is related to the biochemical problem in the brain, whereas CCT emphasizes dysfunction of the psychological process involved in onset of OCD. In addition, the fear of negative events is not a main therapeutic target of the four steps, but it is one in CCT.

There are some limitations to this study. The sample size is relatively small, which reduces the power of the analysis. Also, the preliminary data in the study were obtained from only two institutions. A multicenter trial with independent raters is needed to further determine the efficacy of CCT. The methodology lacks detailed data related to adherence to the psychotherapeutic protocol for CBT. The relationship between adherence and outcome has not been consistently demonstrated (Wampold 2001).

In summary, a more efficacious treatment for OCD is required. Based on the existing knowledge of OCD and our clinical experiences, our study contributes to existing OCD therapies by developing CCT and investigating the efficacy of PCCT for treating OCD. Our preliminary data suggests PCCT has potential for long-term effective treatment of OCD. Further multicenter trials and studies with different cultural backgrounds are needed.
